# Flavor-Enhanced Modulation of Cerebral Blood Flow during Gum Chewing

**DOI:** 10.1371/journal.pone.0066313

**Published:** 2013-06-19

**Authors:** Yoko Hasegawa, Yoshihisa Tachibana, Joe Sakagami, Min Zhang, Masahiro Urade, Takahiro Ono

**Affiliations:** 1 Department of Dentistry and Oral Surgery, Hyogo College of Medicine, Nishinomiya, Japan; 2 Division of Oromaxillofacial Regeneration, Course of Integrated Oral Science, Osaka University Graduate School of Dentistry, Suita, Japan; 3 Division of System Neurophysiology, National Institute for Physiological Sciences, Okazaki, Japan; 4 Department of General Dentistry and Emergency, School of Stomatology, Fourth Military Medical University, Xi’an, China; Tokyo Metropolitan Institute of Medical Science, Japan

## Abstract

**Background:**

Flavor perception, the integration of taste and odor, is a critical factor in eating behavior. It remains unclear how such sensory signals influence the human brain systems that execute the eating behavior.

**Methods:**

We tested cerebral blood flow (CBF) in the frontal lobes bilaterally while subjects chewed three types of gum with different combinations of taste and odor: no taste/no odor gum (C-gum), sweet taste/no odor gum (T-gum), and sweet taste/lemon odor gum (TO-gum). Simultaneous recordings of transcranial Doppler ultrasound (TCD) and near infrared spectrometer (NIRS) were used to measure CBF during gum chewing in 25 healthy volunteers. Bilateral masseter muscle activity was also monitored.

**Results:**

We found that subjects could discriminate the type of gum without prior information. Subjects rated the TO-gum as the most flavorful gum and the C-gum as the least flavorful. Analysis of masseter muscle activity indicated that masticatory motor output during gum chewing was not affected by taste and odor. The TCD/NIRS measurements revealed significantly higher hemodynamic signals when subjects chewed the TO-gum compared to when they chewed the C-gum and T-gum.

**Conclusions:**

These data suggest that taste and odor can influence brain activation during chewing in sensory, cognitive, and motivational processes rather than in motor control.

## Introduction

Taste and odor are critical factors in eating behavior. Taste and olfactory sensory signals are transmitted through chemoreceptors (i.e., taste and odorant receptors), processed in the gustatory and olfactory neural circuits in the central nervous system, and integrated as a sensation of flavor [1], [2]. It is known that taste and odor have significant impact on cognitive and motivational aspects of eating behavior because an inability to taste and smell (e.g., cold, surgical defects, aging) results in loss of pleasure in eating meals [3]–[5]. However, it remains unclear how such sensory signals influence human brain systems involved in eating behavior.

Over the past few decades, a variety of noninvasive measurements, such as functional magnetic resonance imaging (fMRI) and positron emission tomography (PET) have been developed to detect activation in the human brain [6], [7]. In fact, PET and fMRI studies have reported an increase in the cerebral hemodynamic response during chewing [8]–[11]. Although PET and fMRI are powerful tools for examining the neural substrates of behavior with high spatial and temporal resolution, these equipment items require some financial and physical costs (such as long scanning time and radiation damage). Thus, simple bedside techniques need to be developed for easy assessment of brain activation in patients. A near infrared spectrometer (NIRS) is one potential bedside tool that can measure the concentrations of oxygenated hemoglobin (ΔO_2_Hb), deoxygenated hemoglobin (ΔHHb), and total hemoglobin (ΔcHb) [12], [13]. Another method is a transcranial Doppler ultrasound (TCD), which can monitor dynamic changes in cerebral blood flow (CBF) [14]. We have so far reported bilaterally increased CBF during unilateral gum chewing using the TCD method [15]. We have also found that voluntary motor control (e.g., changing the rhythm of jaw movement) is less effective for CBF regulation during chewing [16]. Thus, the present study was designed to investigate how sensory inputs (i.e., taste and odor) could influence the gum-chewing-associated CBF using simultaneous recordings of TCD and NIRS. We found significantly higher CBF during chewing of the gum with taste and odor in comparison to the gum without.

## Materials and Methods

The study protocols were approved by the Ethics Committee of Osaka University Graduate School of Dentistry (No. H21-E31). Each participant approved the written informed consent after the experimenter’s explanation of the aim and methodology of this study.

### 1) Subjects

Twenty-five subjects (13 men and 12 women; 27.0±2.8 y, mean ± SD) with healthy occlusions were enrolled in this study.

### 2) Subjective Ratings of Tested Gums

Subjects tested three types of gum (Lotte, Tokyo, Japan). One type had no taste and no odor (C-gum) as a control gum. One gum had sweet taste only (T-gum). The other gum had sweet taste and lemon odor (TO-gum). We chose these stimuli because the subjects had positive motivations in comparison to the other stimuli such as mint (data not shown). The hardness of each gum was adjusted to the same level. Subjects were instructed to sit on a chair with an eye mask. They were required to chew the different types of gum for 5 min each. The chewing rhythm was regulated by a metronome at the rate of 1 Hz because this frequency required the subjects less efforts [16], while the chewing side was not restricted. The chewing tests were undertaken at least 4 h after meals. A schedule of the tested gums was randomized using a Latin square method. Subjects inserted a piece of gum into their oral cavity immediately before the tests without any information of tested gums. The gum was removed after each test. Breaks of >5 min were interleaved between the tests. After completing all the tests for three types of gum, each subject was required to rate the taste and odor for individual gums by Visual Analog Scales (VAS). Each score ranged from 0 (worst) to 100 (best).

### 3) Brain Hemodynamic Responses

#### 3-1) TCD monitoring

A TCD system (Multi Dop T, DWL, Sipplingen, Germany) was used to bilaterally measure the middle cerebral artery blood flow velocity (MCAV), as described elsewhere [14]. To measure the MCAV, two 2-MHz ultrasonic probes were attached to the subject on upper anterior parts of the bilateral auricles with a head frame (Marc 600, Spencer Technologies, Seattle, WA, USA). Immobility of the probes was confirmed by testing simple jaw closing/opening of the subject. Axial width of the sample volume was set as 10 mm, while the sample depth was set as 48–60 mm. Sampling rate of the TCD was 1 kHz.

#### 3-2) NIRS monitoring

A two-channel NIRS (NIRO-200, Hamamatsu Photonics, Hamamatsu, Japan) was used to monitor the changes in hemoglobin concentration in the left and right frontal lobes. The optode of NIRS consisted of a semiconductor laser as a light source and its light detector, 4 cm apart from each other. This device can measure the tissue absorbance of light at three wavelengths (780, 805, and 830 nm) to detect changes in the concentration of ΔO_2_Hb, ΔHHb, and ΔcHb, respectively [12], [13]. Two optodes were bilaterally attached to the skin over the frontal skull 2 cm from the midline and 1.5 cm over the eyebrow. The sampling rate of the NIRS was 1 Hz.

### 4) Masseter Muscle Activity

Electromyograms (EMG) of bilateral masseter muscles were measured to monitor orofacial motor output during gum chewing [16], [17]. Surface electrodes were attached to the skin over the masseter. The analog signals were amplified by a bio-amplifier (BA-1008, TEAC, Tokyo, Japan), and stored in a personal computer for offline analysis. The sensitivity, time constant, and high-pass filter of the amplifier were set as 100 µV, 0.03 sec, and 3 kHz, respectively. Each subject was asked to clench their jaw with maximum power for 2 sec to calculate the Maximum Voluntary Contraction (MVC). Masseter muscle activity during gum-chewing tests was calculated as the relative value of the MVC.

### 5) Data Analysis

The VAS data for the subjective values of taste and odor were compared among three different gums by repeated measures one-way ANOVA, correcting for multiple comparisons using the Bonferroni method (P<0.017 = 0.05/3). All of the MCAV data were downsampled to 1 Hz sampling by averaging 1,000 data points. The MCAV data during and after the test were accessed by relative values of the averaged MCAV measured during the 5-min rest period before the test. The reference values for ΔO_2_Hb, ΔHHb, and ΔcHb were conventionally defined as the values measured just prior to the test. To test the time course of MCAV, ΔO_2_Hb, ΔHHb and ΔcHb, we calculated the segmental values by averaging the individual data for 1 min, and made statistical comparisons by a repeated measures one-way ANOVA for multiple comparisons using the Bonferroni method (pre-value vs. five during- and five post-values; P<0.005 = 0.05/10). To investigate the effects of taste and odor on individual data (MCAV, ΔO_2_Hb, ΔHHb, ΔcHb, and masseter EMG), we further calculated 5-min averaged data obtained during the tests, and performed statistical comparisons across the time course (i.e., pre-, during-, and post-test values) by a repeated measures one-way ANOVA for multiple comparisons using the Bonferroni method (P<0.017 = 0.05/3).

## Results

**Figure 1:**
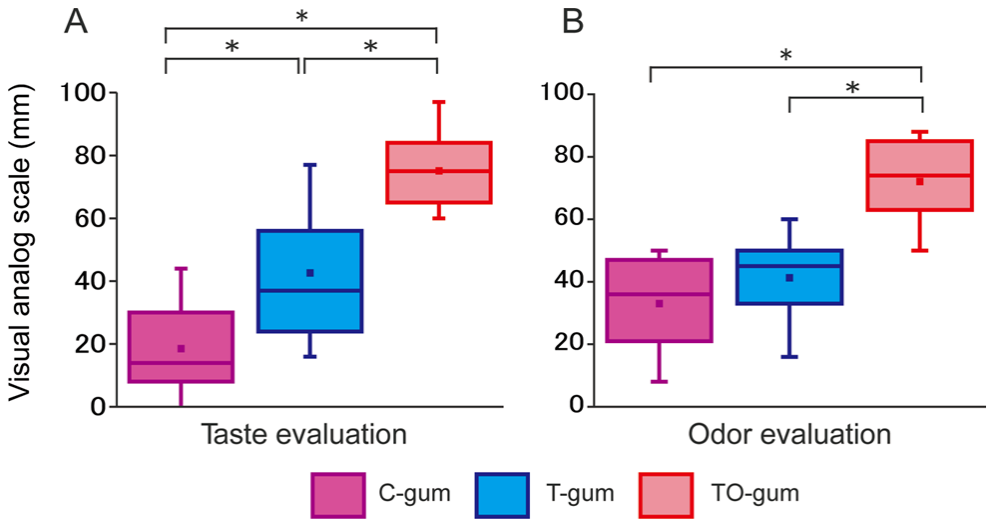
Summaries of Visual Analog Scales (VAS) of taste (A) and odor (B) of tested gums (N = 25). Each scale ranged from 0 (worst) to 100 (best). The boxes are constructed with the top line bounding the first-quartile and the bottom line bounding the third quartile. The horizontal line and the dot in the box indicate the median and mean values, respectively. The short horizontal lines show the largest and smallest values. **P<0.01, ***P<0.001. C-gum (purple): the gum with no taste and no odor (control), T-gum (blue): the gum with the sweet taste only, and TO-gum (red): the gum with sweet taste and lemon odor.

Subjective estimates of taste and odor of tested gums are shown in Figure 1 (N = 25). Subjects’ ratings of the taste of the three types of gum differed significantly (Fig. 1A). The TO-gum had the highest rating for the best taste, while the C-gum had the worst taste rating. The T-gum was intermediate. With regard to the odor, subjects rated the TO-gum the highest. The TO-gum rating was significantly higher than the C-gum and the T-gum rating (Fig. 1B). These data suggest that subjects could discriminate the type of gum tested without prior information.

**Figure 2:**
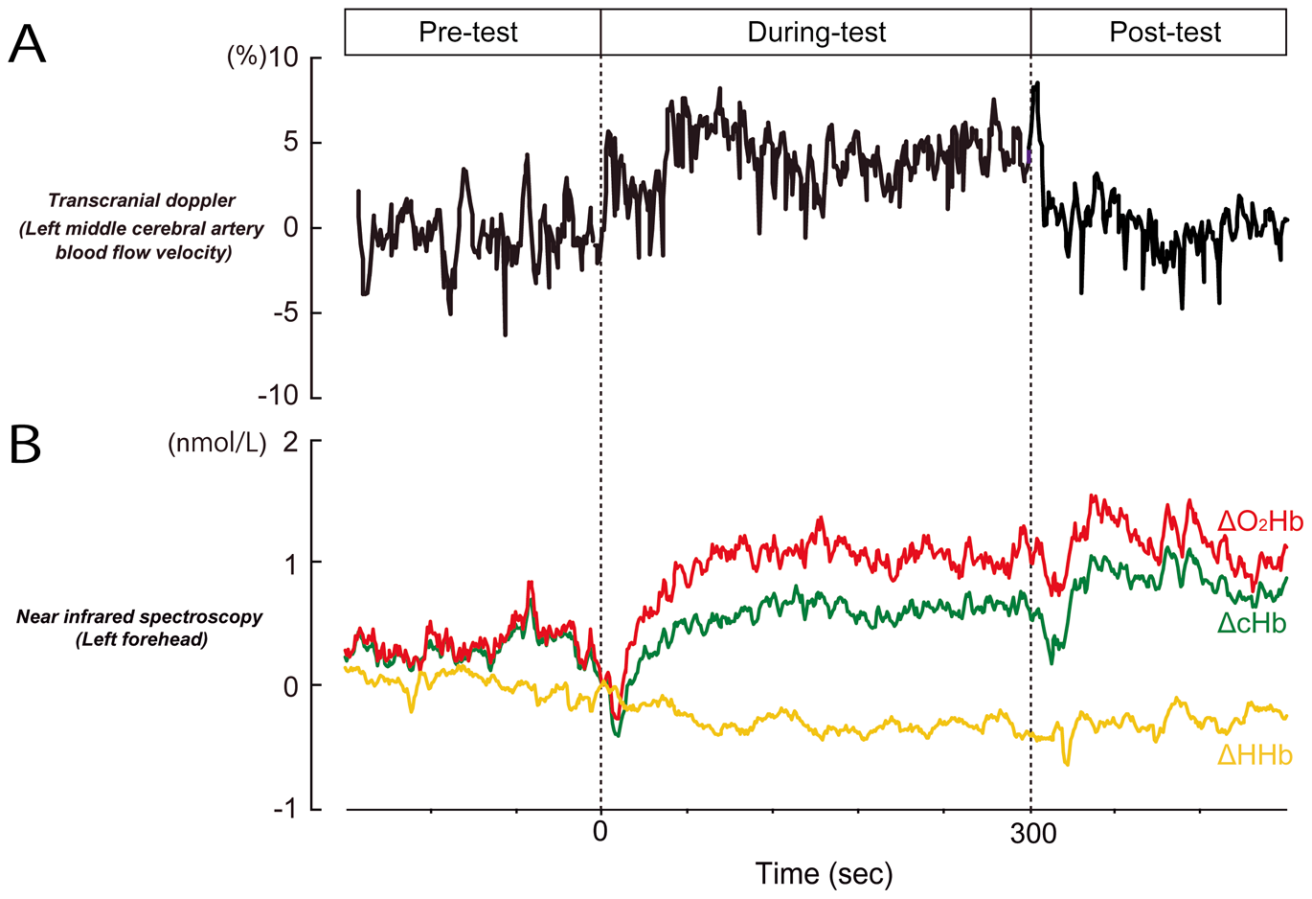
The cerebral hemodynamic changes, associated with TO-gum chewing, simultaneously measured by Transcranial Doppler Ultrasound (TCD) (A) and near infrared spectroscopy (NIRS) (B). Both hemodynamic signals were measured from the left hemisphere. In (A), middle cerebral artery blood flow velocity (MCAV) is shown. In (B), oxygenated hemoglobin (ΔO_2_Hb) is represented by a red line, deoxygenated hemoglobin (ΔHHb) by a yellow line, and total hemoglobin (ΔcHb) by a green line.

Figure 2A shows an example of MCAV in the left hemisphere during TO-gum chewing. The MCAV showed a drastic increase immediately after the onset of chewing, and an immediate decrease after the end of chewing. In Figure 2B, a simultaneously measured ΔO_2_Hb reached to a maximum level 2–3 min after the onset of chewing. The sustained increase lasted more than 5 min after the end of chewing. By contrast, ΔHHb showed a decrease during chewing that outlasted the post-chewing period. Since ΔcHb was a summation of ΔO_2_Hb and ΔHHb, the change in ΔcHb was less obvious than the change in ΔO_2_Hb.

**Figure 3:**
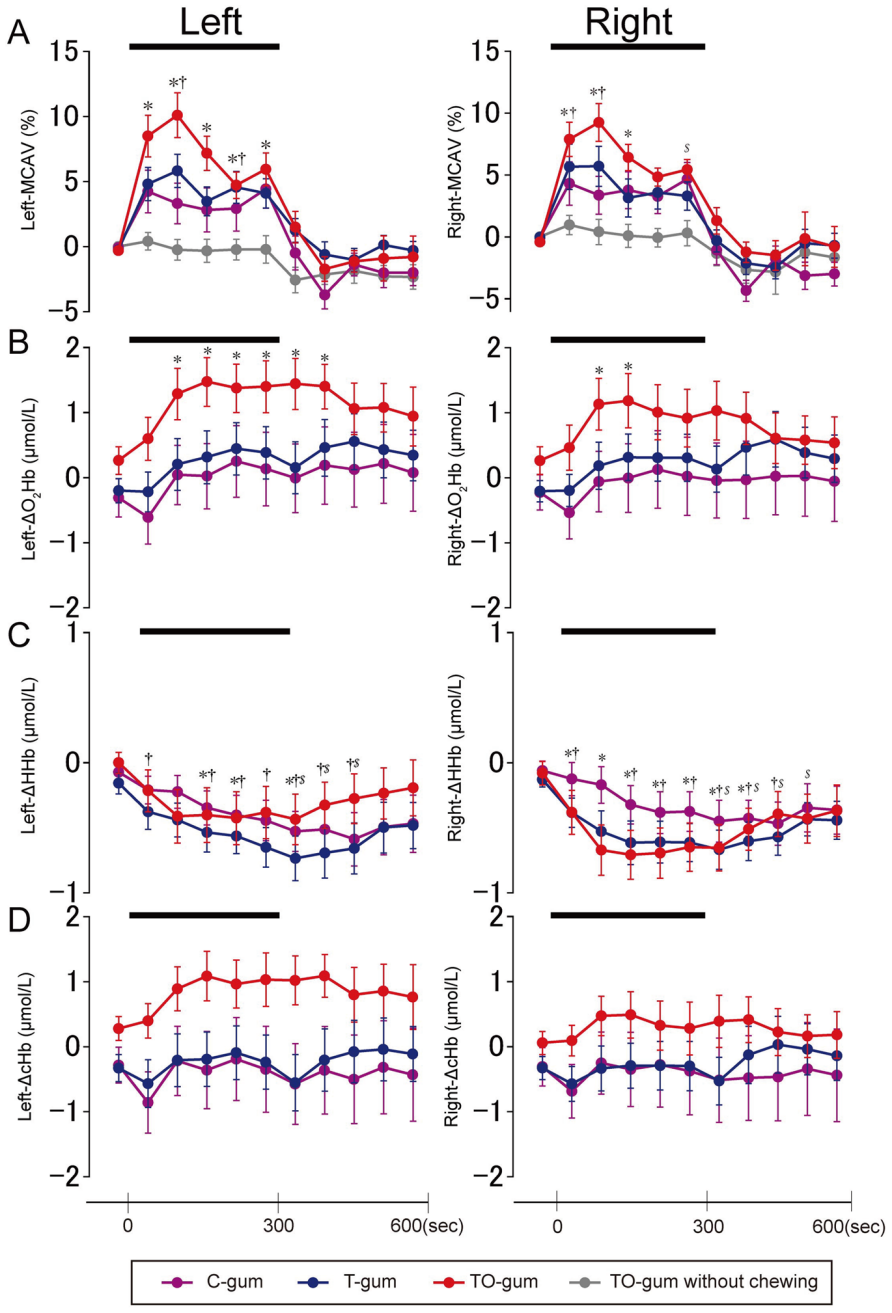
Temporal profiles of averaged (N = 25) hemodynamic changes in the left and right hemispheres measured by TCD (A) and NIRS (B–D) during chewing of three different gums. In (A), the data obtained during putting the TO-gum on the tongue are also superimposed as gray lines. In the NIRS signals (B–D), ΔO_2_Hb is shown in (B), ΔHHb in (C), and ΔcHb in (D). Data are represented as mean ± SEM every 1 min. A bold bar in each panel indicates the duration of the gum-chewing test. Statistical comparisons were performed between the control value (i.e., before chewing) and the value measured at each time point. Significant differences are represented by individual symbols: *, control vs. TO-gum; ^†^, control vs. T-gum; ^$^, control vs. C-gum. Statistically significant levels were set at P = 0.005 ( = 0.05/10) by one-way ANOVA and Bonferroni's correction for multiple comparisons.

Figure 3 shows the time course of averaged data in the TCD-measured MCAV and in the NIRS-measured ΔcHb, ΔO_2_Hb, and ΔHHb. The group data also demonstrated that the left and right MCAV (Fig. 3A) during gum chewing increased depending on the types of gum (see below for additional details). A minute-by-minute change in the MCAV was most obvious when subjects chewed the TO-gum, and least obvious with the C-gum. We further tested the “pure” sensory impact of taste and/or odor on the brain activation by comparison between the MCAV during chewing and the one without chewing. Putting the TO-gum on the tongue did not increase the MCAV (indicated by gray lines in Fig. 3A), suggesting that the flavor-associated brain activation was enhanced by motor execution (chewing behavior).

Regarding the NIRS-measured hemodynamic changes, the bilateral ΔO_2_Hb (Fig. 3B) significantly increased 2 min after the onset of TO-gum chewing, and the sustained activation of the left ΔO_2_Hb was observed for 2 min after chewing ended. No significant increases in the ΔO_2_Hb were observed during T-gum and C-gum chewing, although some post-chewing elevations were observed. On the other hand, the ΔHHb (Fig. 3C) showed small but significant decreases, typically in the late stage of chewing and just after the chewing of any of the gum types. Similar to the example shown in Fig. 2, the group changes in ΔcHb (Fig. 3D) were less obvious than those in ΔO_2_Hb. Since the chewing side was not restricted in this study, similar observations were found in the bilateral ΔO_2_Hb, ΔHHb, and ΔcHb.

**Figure 4:**
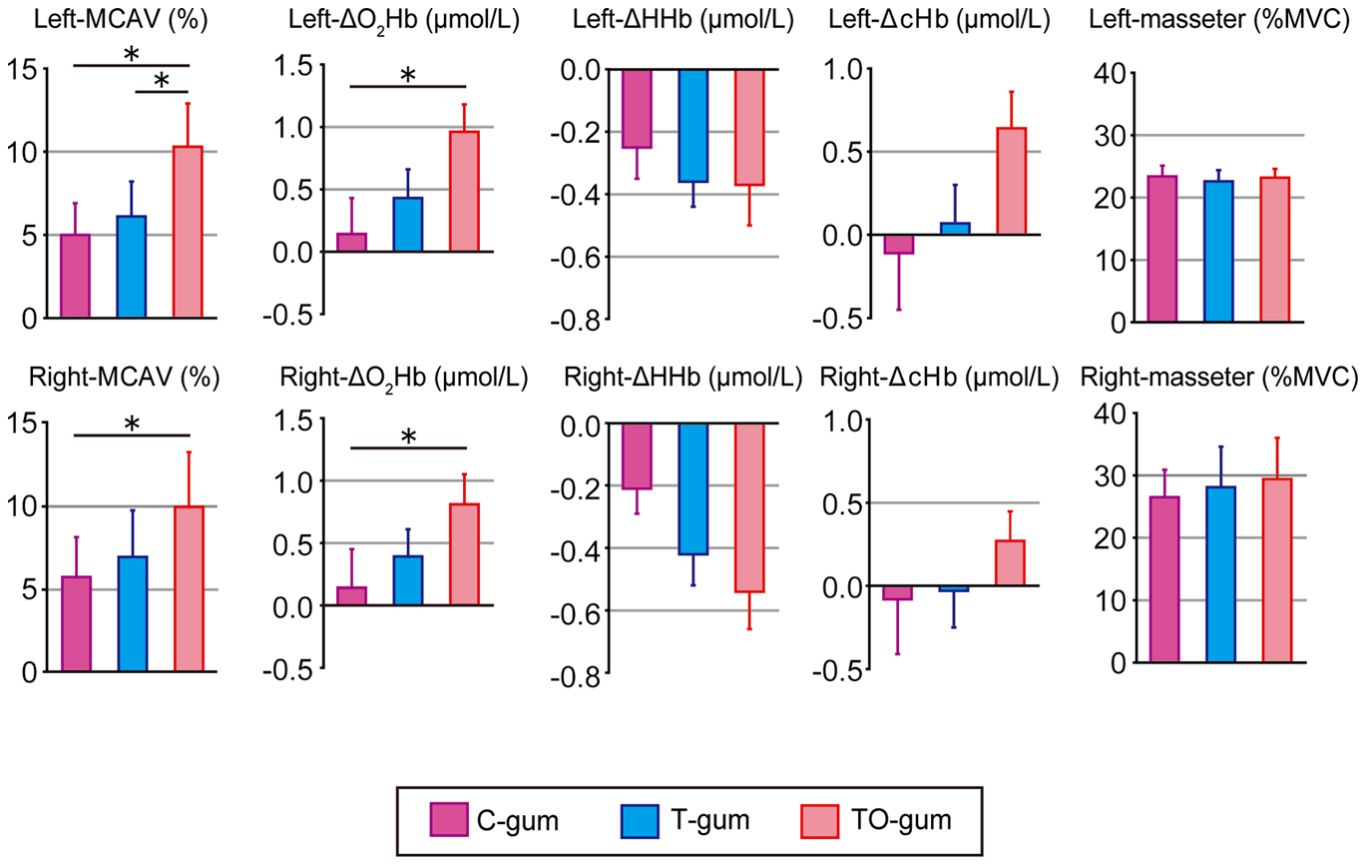
Summaries of hemodynamic changes in the left (top panels) and right (bottom panels) cerebral hemispheres and masseter EMG activity associated with chewing of three different types of gum. In the hemodynamic changes, MCAV, ΔO_2_Hb, ΔHHb, and ΔcHb are arranged from the left to the right. Individual data (mean ± SEM) are represented as change rates of the responses between gum-chewing tests (5 min) and before the test. Statistical comparisons were performed among the three gums. *P<0.05, **P<0.01.

Figure 4 illustrates the summaries of chewing-associated hemodynamic changes in bilateral hemispheres among three different gums. Compared with the C-gum chewing, the TO-gum chewing induced significant increases in the bilateral MCAV and ΔO_2_Hb. By contrast, no significant differences were observed in ΔHHb and ΔcHb. We found no clear differences in the motor output associated with gum chewing (evaluated by masseter EMG) among the three different gums.

## Discussion

The results from this study have demonstrated that the TCD and NIRS are simple but effective bedside tools that can assess chewing-associated brain activation in human subjects. The gum chewing-associated CBF was enhanced by taste and odor. Analysis of masseter EMG activity further revealed that the hemodynamic responses during chewing were not strongly influenced by motor outputs, but rather by sensory, cognitive, and motivational processes in eating behavior.

### Methodological Considerations

We previously established that the TCD is a powerful non-invasive tool to evaluate chewing-associated blood flow in the middle cerebral artery [15]–[17]. The middle cerebral artery supplies a wide variety of cortical areas, such as the primary motor cortex, primary somatosensory cortex, part of the premotor cortex, and subcortical areas including the basal ganglia and thalamus [18]. On the other hand, the NIRS has been developed for non-invasive assessments of hemodynamic change in CBF and cerebral oxygen consumption [12], [13]. In this study, we put the NIRS probe over the skull covering the anterior part of frontal lobes for the following reasons: 1) the TCD probe with a head frame was attached to the upper anterior parts of the bilateral auricles [19] (the NIRS probe should be placed away from the TCD system); and 2) the goal of this study was to detect the sensory, cognitive, and motivational aspects of the taste and odor processing in the brain. The non-motor brain functions are thought to be processed in frontal cortical areas such as the medial and lateral prefrontal cortices, orbitofrontal cortex, and the frontal pole [20], [21]. While the TCD can detect the CBF in the big superficial artery such as middle cerebral artery, this measurement would not be suitable for monitoring the CBF in the deep artery such as anterior cerebral artery covering the medial and orbital prefrontal cortices. Thus, the NIRS probe was centered on the prefrontal areas in the present study. In this regard, the brain areas reflecting the hemodynamic change in the NIRS might be inconsistent with those in the TCD. However, in both methods, the chewing-associated changes in hemodynamic responses were observed depending on whether the taste and odor were present. This indicates that both taste and odor modulate hemodynamic responses in large regions of the brain, affecting sensory, cognitive, and motivational behaviors. Consistent with these data, our previous TCD study reported that the CBF was not influenced by motor parameters such as chewing side and chewing rhythm [16].

### Time Profiles of CBF and NIRS Signals

Neuronal activity in a focal brain area is associated with the regional CBF [22]. An increase in the CBF results in a focal hyperoxygenation. Thus, NIRS can reflect enhanced neuronal activity by measuring an increase in the ΔO_2_Hb concentration accompanied by a decrease in the ΔHHb concentration [23]. Similar to a previous NIRS study, our data demonstrated that the ΔO_2_Hb was most largely changed. Simultaneous measurements of NIRS and PET, using a similar setup as the one used in the present experiment, have indicated that the NIRS signal is most positively correlated with the PET signal measured in the cortical region ranging from 4.5 mm and 13.5 mm from the cortical surface [24], [25]. This means that the ΔO_2_Hb in our NIRS signals could detect the cortical activation successfully. A remarkable feature of the ΔO_2_Hb is that the changes in ΔO_2_Hb lagged behind those in MCAV (Figs. 2 and 3). The delayed response in NIRS might be caused by an instantaneous decrease in the regional CBF secondary to the blood flow to masticatory muscles [26]. The decrease was typical in the smaller vessels and thus more clearly observed in the NIRS signals but not in MCAV signals. The sustained activation after the subjects had stopped chewing may also be due to the difference in the diameter of the measured vessels (i.e., smaller vessels in NIRS vs. larger vessels in MCAV).

### Neural Circuits Involved in the Perception of Taste and Odor

The results from VAS analysis demonstrated that the subjects rated the TO-gum the highest, the T-gum was scored as intermediate, and the C-gum was rated lowest. This means that the subjects could discriminate the type of chewing gum, and had different cognitive and motivational values without prior information. The olfactory sensation of the gum is processed in the pathway from the olfactory epithelium, the olfactory bulb, to the olfactory cortex (piriform cortex) [27]. On the other hand, the taste sensation is processed in the pathway from the taste bud, the solitary nucleus, the parabrachial nucleus, the thalamus, to the gustatory cortex (frontal operculum and insula) [28]. The processed information in the primary olfactory and taste cortices are integrated in higher brain areas such as orbitofrontal cortex and amygdala. These regions then give rise to the perception of the flavor [29]. Our data demonstrated that the hemodynamic changes in TCD and NIRS were bigger in TO-gum than in T-gum. This indicates that the frontal brain activation may occur more strongly in the process of multimodal integration than in unimodal processing.

### Clinical Implications

Our data indicate that a combination of NIRS and TCD can be powerful bedside tools to monitor the chewing-associated hemodynamic changes in wide areas of frontal brain regions. In fact, the comprehensive measurements can detect sensory, cognitive, and motivational impact of taste and odor on brain activation during chewing. Importantly, our results demonstrated that chewing the flavored gum increased the brain activation while putting the gum on the tongue did not. This suggests that the sensorimotor integration/transformation may be more important to induce the brain activation than the “pure” sensory processing of taste and odor. Furthermore, eating is an essential behavior that can induce positive emotions and vigor in daily life. In elderly people, keeping the ability to taste and smell during eating may contribute to neuronal activation in wide areas of the cerebral cortex and subcortical structures. The NIRS and TCD could also be simple but effective tools for the assessment of abnormal processing of taste and odor in patients with central and peripheral neurological disorders.
