# Correction: Flavor-Enhanced Modulation of Cerebral Blood Flow during Gum Chewing

**DOI:** 10.1371/annotation/d1f70d63-1261-49c1-9499-6ef5763a6c90

**Published:** 2013-10-09

**Authors:** Yoko Hasegawa, Yoshihisa Tachibana, Joe Sakagami, Min Zhang, Masahiro Urade, Takahiro Ono

Multiple funding organizations and grants were incorrectly omitted from the Funding Statement. 

The Funding Statement should read: "This study was supported by KAKENHI from the Ministry of Education, Science and Culture of Japan (Grant No. 21791887, No. 23792263), Grant-in-Aid for Scientific Research on Innovative Areas (No. 24120522), the Society for Research on Umami Taste, and the Global COE Program in "In Silico Medicine" of Osaka University. The authors declare no conflict of interest with any financial organization regarding the manuscript. The funders had no role in study design, data collection and analysis, decision to publish, or preparation of the manuscript."

